# Accelerated 2D Classification With ISAC Using GPUs

**DOI:** 10.3389/fmolb.2022.919994

**Published:** 2022-07-06

**Authors:** Fabian Schöenfeld, Markus Stabrin, Tanvir R. Shaikh, Thorsten Wagner, Stefan Raunser

**Affiliations:** Department of Structural Biochemistry, Max Planck Institute of Molecular Physiology, Dortmund, Germany

**Keywords:** 2D classification, GPU, CUDA, cryo-EM, SPHIRE, 2D class averages

## Abstract

A widely used approach to analyze single particles in electron microscopy data is 2D classification. This process is very computationally expensive, especially when large data sets are analyzed. In this paper we present GPU ISAC, a newly developed, GPU-accelerated version of the established **I**terative **S**table **A**lignment and **C**lustering (ISAC) algorithm for 2D images and generating class averages. While the previously existing implementation of ISAC relied on a computer cluster, GPU ISAC enables users to produce high quality 2D class averages from large-scale data sets on a single desktop machine equipped with affordable, consumer-grade GPUs such as Nvidia GeForce GTX 1080 TI cards. With only two such cards GPU ISAC matches the performance of twelve high end cluster nodes and, using high performance GPUs, is able to produce class averages from a million particles in between six to thirteen hours, depending on data set quality and box size. We also show GPU ISAC to scale linearly in all input dimensions, and thereby capable of scaling well with the increasing data load demand of future data sets. Further user experience improvements integrate GPU ISAC seamlessly into the existing SPHIRE GUI, as well as the TranSPHIRE on-the-fly processing pipeline. It is open source and can be downloaded at https://gitlab.gwdg.de/mpi-dortmund/sphire/cuISAC/

## 1 Introduction

Since the “resolution revolution” (Kühlbrandt, 2014), single particle electron cryomicroscopy (cryo-EM) has established itself as a prime tool for determining the three dimensional structure of macromolecular complexes at high resolution. Over time, this success has motivated the development of novel technologies that fuel a continuous trend of producing ever larger data sets in ever shorter amounts of time. Consequently, the software developed to process cryo-EM data sets has to continuously evolve in order to keep up with the computational demand of this trend.

Here we focus on 2D classification, a crucial step when processing cryo-EM data sets for single particle analysis (*SPA*). During 2D classification, a stack of particles is sorted into different subsets that have been determined by the classifier based on apparent similarities. In conjunction with this class assignment, 2D classification also produces a set of 2D alignment parameters per particle. These alignment parameters are applied to the individual particles of a class in order to bring them into register with each other and form a single average for each class. The resulting set of class averages produced by 2D classification serves two distinct purposes: 1) 2D class averages are usually the first visual result produced when processing a cryo-EM data set and, consequently, serve as a critical early indicator about the overall quality of a new data set, as well as its potential to yield a high-resolution three-dimensional reconstruction. As data sets continue to rapidly increase in size, the required computational power to process them equally rises. Since computational capacity is usually limited, it is therefore essential for experimentalists to be able to quickly accept or dismiss a new data set for further processing and/or change the parameters of data acquisition—ideally while data acquisition is still ongoing. 2) Class averages further depict the internal consistency of a sample, i.e., whether the data includes different conformational states of the target protein, unwanted background proteins or damaged particles. Once classified, such undesired elements can easily be excluded from a data set. This not only increases the signal to noise ratio (SNR) of the data set and thus allows for a higher resolution reconstruction, but also shortens the required processing time due to the reduced size of the data set. In addition, 2D class averages are also used in some cryo-EM processing suites to produce an initial 3D reconstruction of the targeted macromolecular complex, e.g., SPHIRE (Moriya et al., 2017).

2D classification is a computationally expensive procedure that is routinely used in processing cryo-EM data. *Inter alia* it is confronted with the following challenges: The low signal-to-noise ratio (SNR) inherent to cryo-EM data sets; the ability to cross-reference an ever-increasing number of images; and an uneven distribution of particles that should be represented within the final class averages. As a consequence different strategies are being employed by the classifiers of different software packages to overcome these issues (Frank et al., 1996; Hohn et al., 2007; Tang et al., 2007; Scheres, 2012; Punjani et al., 2017).

Of these, **I**terative **S**table **A**lignment and **C**lustering (ISAC) is an established 2D clustering algorithm that employs repeated rounds of clustering coupled with a stability metric for each newly found cluster (Yang et al., 2012). ISAC has been widely used for processing cryo-EM data and contributed to multiple high-resolution reconstructions (Schubert et al., 2018; Roderer et al., 2019; Pandey et al., 2021; Zhang et al., 2021; Zhao et al., 2021). However, while ISAC has been received with widespread acclaim, its use incurs an often prohibitively high computational cost. Indeed, running ISAC requires a full scale computer cluster, where execution is usually distributed across hundreds of CPU cores.

Consequently, we present GPU ISAC: An extended new version of the existing ISAC algorithm where the primary computational bottlenecks have been outsourced on any available GPUs. In conjunction with new internal data batching mechanisms, GPU ISAC allows users to perform ISAC 2D classification on a single computer outfitted with one or more consumer-grade GPUs. This markedly improves the accessibility of ISAC, as users may simply run the clustering on their own machines without having to manage a remote queuing system, and/or a computing cluster. This heightened accessibility further translates into enhanced flexibility, as shorter runs can now be executed more freely on single machines in order to identify suitable hyperparameter settings, or simply produce early class averages as soon as the data become available during acquisition. Other than foregoing the need for an expensive computer cluster, the ability of GPU ISAC to run on a single machine also greatly simplifies the integration of ISAC-quality clustering into other workflows. This has been demonstrated in TranSPHIRE (Stabrin et al., 2020), where GPU ISAC is one of the key tools enabling on-the-fly cryo-EM processing.

## 2 Materials and Methods

The previous implementation of the original ISAC algorithm (Yang et al., 2012) was modified and sped up by the original authors, called ISAC2, and is included in the SPHIRE package (Moriya et al., 2017; Wagner et al., 2021). This work is based on ISAC2, which depends on the computational power of a computer cluster, where data processing is distributed across the numerous CPU cores of multiple machines using MPI [Message-Passing Interface (Lusk and Gropp, 1995)]. Access to a computer cluster, however, is often a prohibitive requirement: Acquisition and maintenance of a local cluster are costly endeavors, while accessing an existing, remote cluster usually implies dealing with queuing systems and tedious waiting times. Use of a remote cluster also requires the transfer of (large) cryo-EM data sets, and computationally expensive cryo-EM jobs are often scheduled to yield their time to smaller jobs that can be finished faster.

GPU ISAC was specifically developed to forego this requirement and enable users to run ISAC 2D clustering on a single machine. To do so, GPU ISAC employs any locally available graphics cards as co-processors for addressing the computational bottlenecks of ISAC2. In addition to this new GPU parallelization, GPU ISAC retains the original MPI parallelization of ISAC2. However, multi-node processing is not yet supported. The resulting hybrid parallelization allows GPU ISAC to make use of both the available GPU and CPU processing power. In order to distinguish between these two frameworks below, we differentiate between “processes” and “threads”: The MPI parallelization distributes its computation across “processes” (running on individual CPU cores), while the GPU parallelization distributes its computation across “threads” running on the cores of a graphics card. Note that a graphics card holds many graphical processing units (GPUs) but we nevertheless refer to the whole graphics card as “one GPU” as a shorthand. The GPU code itself is implemented in C++ and uses the Nvidia CUDA framework to execute parallel code (“kernels”) on the physical GPUs (Nickolls et al., 2008). The Python ctypes library is used to communicate between the Python implementation and the C++/CUDA code.

Profiling the execution of ISAC2 reveals the primary computational bottlenecks to be the numerous 2D alignment functions used to ensure the stability and reproducibility of the ISAC algorithm. At its core, an alignment function f_aln_ (**p**_i_,**r**_j_) aims to find the geometric transformation required to bring a given particle **p**_i_ into register with a reference **r**_j_. To do so, particle **p**_i_ can be thought of as moving along the x- and y-axis within a certain search range [s_min_, s_max_] × [s_min_, s_max_], and rotated by 360 degrees at each position. For each permutation of shift and rotation, the cross-correlation of the modified particle and the reference is computed. This process is repeated for a mirrored version of **p**_i_. The alignment function then searches for the highest found cross-correlation value and returns a tuple

$${\text{f}}_{\text{aln}}\left(p_{\text{i}},r_{\text{j}}\right)=y_{\text{ij}}=\left({\text{s}}_{\text{ijx}},{\text{s}}_{\text{ijy}},\theta_{\text{ij}},{\text{m}}_{\text{ij}}\right)$$

of alignment parameters, where s_ijx_ ∈ [s_min_, s_max_] and s_ijy_ ∈ [s_min_, s_max_] denote shifts in x- and y-direction, respectively; θ_ij_ ∈ [0, 360] denotes angle of rotation; and m_ij_ ∈ [0,1] denotes whether the particle needs to be mirrored. Applying the transformation described by the alignment parameters stored in tuple **y**_ij_ to particle **p**_i_ brings particle **p**_i_ into register with reference **r**_j_.

In the ISAC2 CPU-only framework, the above described alignment function f_aln_ (**p**_i_,**r**_j_), conceptually, is implemented as follows: Particle **p**_i_ is shifted by each combination of shifts within the [s_min_, s_max_] × [s_min_, s_max_] search range. Both **p**_i_ and **r**_j_ are then ring-wise re-sampled into a polar representation. Each ring of these polar-converted images is then transformed by a fast Fourier transformation (FFT), and the resulting 1D FFTs are multiplied to compute their cross-correlation (Yang et al., 2012). The result of this multiplication is a vector **ccf**_ij_ containing the cross-correlation values for reference **r**_j_ and all rotated versions of particle **p**_i_. The increment in angle per data point in **ccf**_ij_ is determined by the number of sample points per ring during the polar conversion step. The best rotational match to align **p**_i_ and **r**_j_, given the used shift values, can then be inferred from the position of the maximum value in **ccf**_ij_.

### 2.1 GPU Framework

**FIGURE 1:**
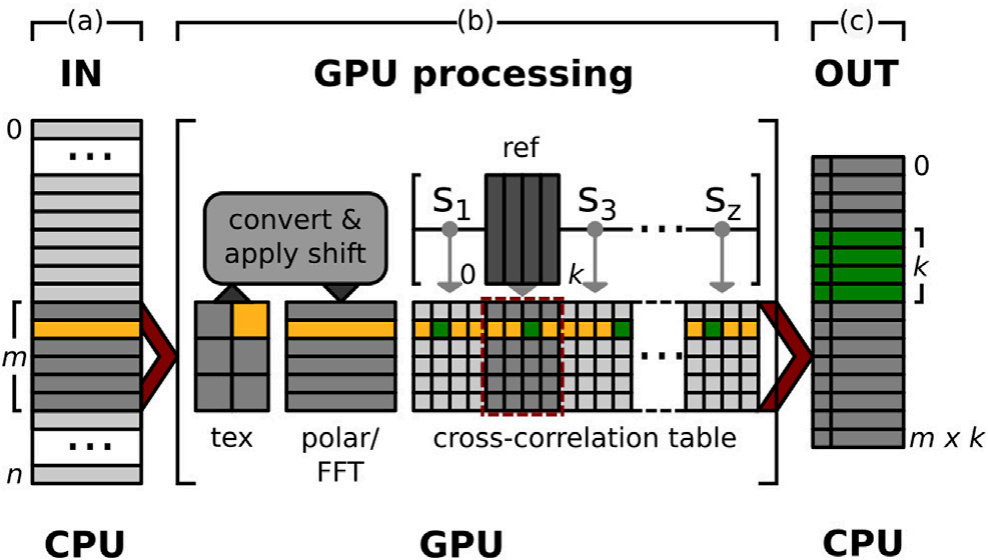
GPU alignment pipeline. Pipeline to compute the alignment parameters to bring a stack of *n* particles into register with *k* references, each. To help illustrate the overall process, the memory used by the same image throughout the different memory buffers of the pipeline is highlighted in yellow. The memory to store the computed alignment parameters of this particle and all references are marked in green. **(A)** IN: Stack of *n* input particles. The pipeline processes substacks of size *m* (dark grey) of the overall data stack until all data is processed. **(B)** GPU processing: The images of the next substack to be processed are first transferred to the GPU and stored in CUDA texture memory (**tex**). A CUDA kernel then converts all images to their polar representation while simultaneously applying a translational shift s_l_ ∈ [s_0_, ..., s_z_] and normalization. Furthermore an FFT is applied to each image (**polar/FFT**). Next, to compute the cross-correlation, a second CUDA kernel multiplies the *m* transformed particles with *k* references. The results are stored in a cross-correlation table where the *i*-th row contains the cross-correlation results of particle **p**_i_ with all *k* references. A final CUDA kernel searches row_i_ for one maximum per reference and computes the alignment parameters used to produce each of the *k* maxima in row_i_. **(C)** OUT: Alignment parameters for the *m* particles of the last input substack. For each particle **p**_i_ we compute *k* alignment parameter tuples to best align **p**_i_ with each of the *k* references. This results in an output stack of *m* x *k* alignment parameter tuples per substack.

In GPU ISAC, the alignment is implemented using a series of parallel CUDA kernels that are executed on the available GPUs of the local machine. These kernels implement the following modularized functions: conversion into a polar representation; image normalization; image transformation according to given alignment parameters; cross-correlation computation; maximum search per class; and extraction of alignment parameters. Taken together, these kernels offer a full software suite to perform 2D alignment on the GPU (Figure 1). The necessary memory buffers are allocated by a separate initialization function beforehand, in order to allow multiple 2D alignment calls to be executed in rapid succession without the need to repeatedly re-initialize memory buffers on the GPU. We make use of CUDA unified memory to create buffers that can be accessed by code running on the GPU as well as on the CPU, which greatly simplifies providing the kernels with their input and retrieving their output. The addresses to these buffers are communicated to Python using the ctypes library, allowing Python direct access to memory filled by CUDA kernels. This memory layout enables the straightforward embedding of kernel-side GPU computations into the Python source without the need to employ an additional library to manage GPU access. Instead, all top-level GPU functionality to perform 2D alignment is provided in the form of functions directly callable from within Python.

**FIGURE 2:**
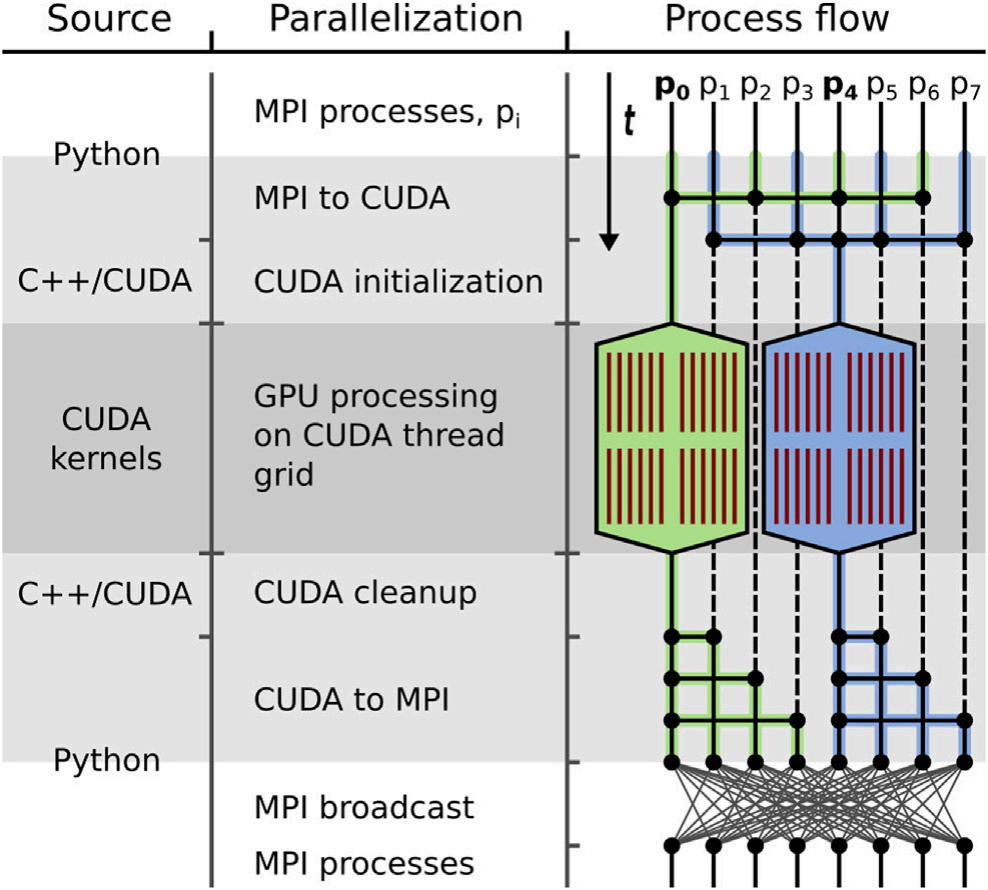
GPU processing embedding. General template of outsourcing bottleneck computations to available GPUs in GPU ISAC. In all three columns, time flows from top to bottom. Shown are the different source code layers (left), the different modes of parallelization (center), and the data processing flow (right) while GPU ISAC transitions between data processing on the local CPUs and the local GPUs. Transition times between the CPU/MPI and GPU/CUDA parallelization are highlighted in light grey; GPU processing time is highlighted in dark grey. **Left:** The original ISAC2 implementation is written in Python, with a C++ backend. GPU ISAC employs an intermediate C++ layer to transfer data between the Python code running on the CPU and the CUDA kernels running on the GPU. **Center:** Embedding GPU processing within the existing MPI parallelization first requires a transition from MPI to CUDA. This includes a re-distribution of data, as well as switching over control from using a small set of MPI processes on the CPU to a large number of threads on the GPU. This translates into initializing all necessary resources on the GPU and executing the parallel CUDA kernels on the GPU to perform the actual computations. Afterwards the program transitions back to the MPI parallelization by having the GPU-controlling MPI processes broadcast their results to all MPI processes. **Right:** From top to bottom, the control flow is depicted as processing switches, in this example, between eight MPI processes and a grid of 48 CUDA threads (red) spread across two GPUs (stretched hexagons). Since two GPUs are being used the overall work load is split into two parts, highlighted in green and blue, respectively. During the MPI to CUDA transition the data distributed across all MPI threads is collected in MPI processes p_0_ and p_4_. These two MPI processes are responsible for GPU initialization, data transfer, and executing the CUDA kernels. While the CUDA kernels are running, the MPI processes remain idle (stippled lines). Once the kernels have stopped, processes p_0_ and p_4_ distribute the data again. Depending on the follow-up needs of the MPI parallelization this either means sending the GPU results to a chosen subset of MPI processes, or performing a global data synchronization *via* MPI broadcast.

While individual GPU alignment functions can be called from within Python, their integration into the existing ISAC2 code requires a transition from the original MPI parallelization to using the GPU-parallelized CUDA kernels. This means that data processing switches from occurring on all MPI processes to only *g* MPI processes, each of which employs one of *g* locally available GPUs for processing. In GPU ISAC this transition is done *via* a multi-step approach: Initially all image data are read into CPU-side RAM and processing is distributed across as many processes as the local CPU can provide. When switching over to data processing using *g* GPUs, the overall work load of *n* particles is split into *g* subsets of size *n*:*g*. We select *g* MPI processes that each collect the data of one such subset and transfer it to an assigned GPU for processing (Figure 2). In this way, the modularized design of our CUDA kernels allows GPU ISAC to easily distribute the overall work load across an arbitrary number of local GPUs. Once the CUDA kernels have completed their computations, their results are found within a Python-readable data array and are re-distributed across all MPI processes *via* MPI broadcasts.

To resolve any 2D alignment bottlenecks we accelerated three different stages of the overall ISAC algorithm: 1) A one-time pre-alignment step to align all images of the input stack with a single, global reference; 2) repeated multi-reference alignment tied to the k-means clustering at the core of ISAC; and 3) repeated multi-class alignment to ensure the stability of the classes determined by the preceding k-means clustering. While the general GPU kernel suite summarized above is applied in all three of these cases, the individual top-level functions are adapted to exploit the particularities of each alignment bottleneck. These C++ functions are called by Python and, in each case, are responsible for initializing GPU resources; packaging the data and feeding it to the different CUDA kernels; move the results into Python-accessible buffers; and finally clear all used GPU memory.

#### 2.1.1 GPU Pre-alignment

Before ISAC can begin the clustering process, the input data is read into RAM and pre-processed. This pre-processing in GPU ISAC has been re-worked to be more efficiently parallelized across CPU processes using MPI in order to be able to process larger data sets on a single machine. As a part of this process a pre-alignment step is called, where all particles of the input stack are aligned to one common, global reference. Alignment with only a single reference translates into a much smaller cross-correlation table on the GPU (Figure 1). The resulting freed up memory allows the pre-alignment to store a larger number of particles on the GPU for parallel processing. The exact number of particles that can be processed on the GPU simultaneously is dynamically determined during runtime by an initialization function specifically written to compute the memory consumption of the pre-alignment procedure. The overall input stack is then subdivided into batches of the determined size and all batches are sequentially distributed among the available GPUs for processing. The pre-alignment iterates 14 times to refine the global average before the final alignment parameters for each particle are collected and written to disk.

#### 2.1.2 GPU Multi-Reference Alignment

Multi-reference alignment takes place during the k-means clustering at the core of ISAC. It is the most generic alignment computation where each of a (large) number of particles is aligned with a (smaller) number of references. For each individual particle **p**_i_ the result of multi-reference alignment is a set of alignment parameter tuples **Y**_i_ = [t_i0_, ..., t_ik_] where tuple t_ij_ stores the parameters to align particle **p**_i_ with class average **r**_j_ ∈ [r_0_, ..., r_k_] (Figure 1). In addition, multi-reference alignment provides a vector containing the cross-correlation values of each particle when aligned with all class averages. These values are used to determine the best matching class per particle, as well as the second best match, etc., while any GPU may easily be able to store all class averages at the same time, the cross-correlation table grows rapidly with the inclusion of additional alignment references. Therefore the overall input particle stack is subdivided into multiple batches, each of which is aligned with a subset of all references. Reference batches are cycled before particle batches, ensuring that no particle is transferred to the GPU more than once when aligned with all references. This order is chosen since cycling repeatedly through the usually much smaller number of references is significantly cheaper than cycling the same number of times through the overall input particle stack.

#### 2.1.3 GPU Multi-Class Alignment

Multi-class alignment is part of the stability test of the ISAC algorithm. Briefly, ISAC employs k-means clustering to determine classes and, after a set number of clustering iterations, performs a “stability test” to confirm the membership of each particle to its assigned class. The stability test includes 30 alignment iterations of all particles with their assigned class averages, and is itself repeated five times (default values). As can be expected, stability testing is one of the primary computational bottlenecks of ISAC. Because stability testing happens after clustering, all particles are assigned their particular class and the concomitant class average (reference) for alignment. This translates into the GPU storing a number of references, but still a reduced cross-correlation table since every particle is only aligned to a single, pre-determined reference (Figure 1). In order to avoid memory transfer overhead, the GPU multi-class alignment does not determine the number of particles that can be processed at the same time on the GPU, but rather the number of fixed-sized classes. Accordingly, the overall data is split into subsets of classes and their associated particles. This allows GPU ISAC to guarantee that every particle is transferred to the GPU only once when computing 30 alignment iterations per class during each stability test iteration. The global work load is therefore split into subsets of classes and again distributed across all available GPUs after ascertaining the available memory at the time.

### 2.2 Hardware

To assess the performance and scaling behavior of GPU ISAC we profiled its run time across different data sets and hardware platforms. We chose three different machines: 1) one machine featuring an Intel i9-7920X CPU and two Nvidia Geforce GTX 1080 TI graphics cards; 2) a second machine featuring an Intel i7-6950X CPU and four Nvidia Geforce GTX 1080 TI cards; and 3) a third machine featuring an Intel i9-7920X CPU and two Nvidia Quadro GV-100 cards. Note that regardless of the actual number of available CPU cores, all testing was done using six CPU cores, translating to six parallel MPI processes. When processing larger data sets (1M+), we used a high performance machine equipped with an AMD Ryzen Threadripper 3990X (32 cores) and two Nvidia Quadro RTX 6000 cards.

### 2.3 Data

For testing and profiling we used a number of different cryo-EM data sets: 1) rigor actomyosin-V complex (Pospich et al., 2021), to represent filamentous structures; 2) hamster sterol regulatory element-binding protein cleavage-activating protein (SCAP) (Weyers, 2021), and 3) zebrafish transient receptor channel 4 (TRPC4) in amphipols (Vinayagam et al., 2020), to represent membrane proteins; 4) myosin-Va-S1 fragment bound to one essential light chain (∼120 kDa), to represent small proteins; 5) a cell adhesion receptor (CAR), an in-house dataset to represent a difficult to process data set and estimate an upper bound for processing, and 6) TdcA1 toxin subunit from Photorhabdus luminescens (Gatsogiannis et al., 2013) (EMPIAR-10089), to represent a well-behaving data set. This data set originally only held 10,000 particles, which were duplicated to artificially produce data sets matching the respective experiment. Since our experiments only evaluate the timing rather than the classification performance, duplicating a dataset is not a concern.

### 2.4 GPU Iterative Stable Alignment and Clustering Parameters

If not specified otherwise, all timing runs use GPU ISAC default parameters. In particular, this means that GPU ISAC will attempt to produce 200 classes and automatically determine the maximum class size from the number of particles in the data set accordingly. The minimum class size is set to 2/3 of the maximum class size. Further, GPU ISAC by default uses only 95% of the available GPU memory in order to leave space for any operating system and/or CUDA library-associated allocations (e.g., the CUDA FFT library CUFFT).

## 3 Results

Thanks to the ability of GPU ISAC to employ locally available graphics cards as co-processors, the ISAC 2D clustering algorithm can now be successfully executed on individual desktop computers. Since the previous CPU-only implementation ISAC2 lacks this ability, we cannot compare the performance of GPU ISAC and ISAC2 directly. We can, however, process identical data sets on a computer cluster to estimate the number of CPU processes that GPU ISAC is able to compensate for. In addition, in order to determine the general performance of GPU ISAC, we processed multiple identical data sets on machines featuring different GPU configurations. Furthermore, we demonstrate the scaling behavior of GPU ISAC when processing an increasing number of particles under different clustering conditions.

### 3.1 General Performance

#### 3.1.1 GPU Iterative Stable Alignment and Clustering Execution Profile

To showcase the GPU ISAC execution profile in general and the performance of the newly developed CUDA kernel specifically, we processed increasingly larger input stacks of a myosin-Va-S1 data set and extracted all relevant time stamps from the automatically generated GPU ISAC log files. Here, as well as in the following, we focus our analysis on the time spent in the three GPU-accelerated bottleneck functions (Supplementary Figures S1A–C) and the overall runtime of GPU ISAC (Supplementary Figure S1D). For more than 200k particles GPU ISAC is increasingly limited by IO (Supplementary Figure S1D **)** and is therefore more dependent on fast data reading. For example, processing 500,000 myosin-Va-S1 particles using two GV-100 cards amounts to about ten hours. Of these, GPU ISAC spends a total of about 3.3 h executing its CUDA kernels where the majority of data processing takes place. The rest of the time is spent on pre-processing, file operation, and data structure maintenance across the processes of the MPI parallelization. In the case of myosin-Va-S1, the default-parameter induced limit of 200 equal-sized classes is almost reached when using 200,000 particles, after which an increase to 500,000 particles only yields a small number of additional classes (Supplementary Figure S1E).

#### 3.1.2 GPU Selection

Since all bottleneck computations are being performed on GPUs, the total number and the type of the available graphics cards are crucial factors for the overall runtime. GPU ISAC will automatically check the amount of available GPU memory and configure itself to process as many particles in parallel as possible, across all available GPUs. Consequently, the more GPUs are available, and the more powerful they are, the faster GPU ISAC will be able to process a given data set. To determine the impact of the selected GPUs on the overall performance of GPU ISAC, we measured the total runtime when processing the same data sets on three different GPU configurations: two GTX 1080 TI graphics cards; four GTX 1080 TI cards; and two GV-100 cards. GPU ISAC was used to process subsets of 10,000, 50,000, 200,000, and 500,000 particles from a myosin-Va-S1 data set. The pre-alignment (Supplementary Figure S2A), multireference alignment (Supplementary Figure S2B), stability alignment (Supplementary Figure S2C), and the total runtime (Supplementary Figure S2D) of GPU ISAC all scale linearly with the input size of the processed data set, and four GTX 1080 TI cards can process 500,000 particles in less than five hours.

#### 3.1.3 Data Set Impact

**FIGURE 3:**
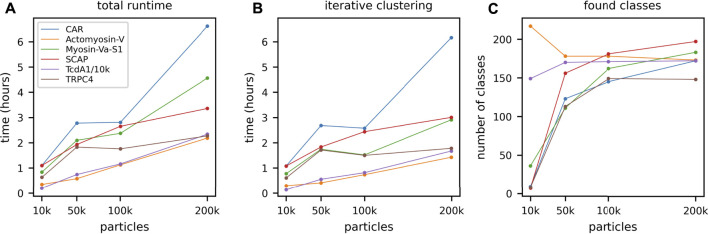
GPU ISAC runtime variance when processing different data sets. To demonstrate the runtime variance across data sets, we processed six different data sets of varying quality and clustering difficulty: Cell adhesion receptor (CAR) **(blue)**, actomyosin-V **(orange)**, myosin **(green)**, SCAP **(red)**, TcdA1 **(purple)**, and TRPC4 **(brown)**. For each data set we processed subsets containing 10,000, 50,000, 100,000, and 200,000 particles, respectively. **(A)** While the runtime of the CUDA kernels scales strictly linearly (Supplementary Figure S1), full-scale GPU ISAC runs display a higher amount of variance in their scaling across data sets. However, it can be seen that the runtime still approximates linear scaling rather than exhibiting quadratic (or worse) scaling behavior. **(B)** The scaling behavior shown for the total runtime is mirrored in the pure processing parts of GPU ISAC (iterative clustering). For up to 200k particles GPU ISAC is not IO limited and spends the majority of its time in the actual data processing, rather than data handling. **(C)** While the number of produced classes across different runs and data sets rises when more data is used this is not a linear trend. While the default parameters impose an upper limit of 200 found classes, it can be seen that this limit is not reached in all cases and simply using more particles does not necessarily yield significantly more averages.

**FIGURE 4:**
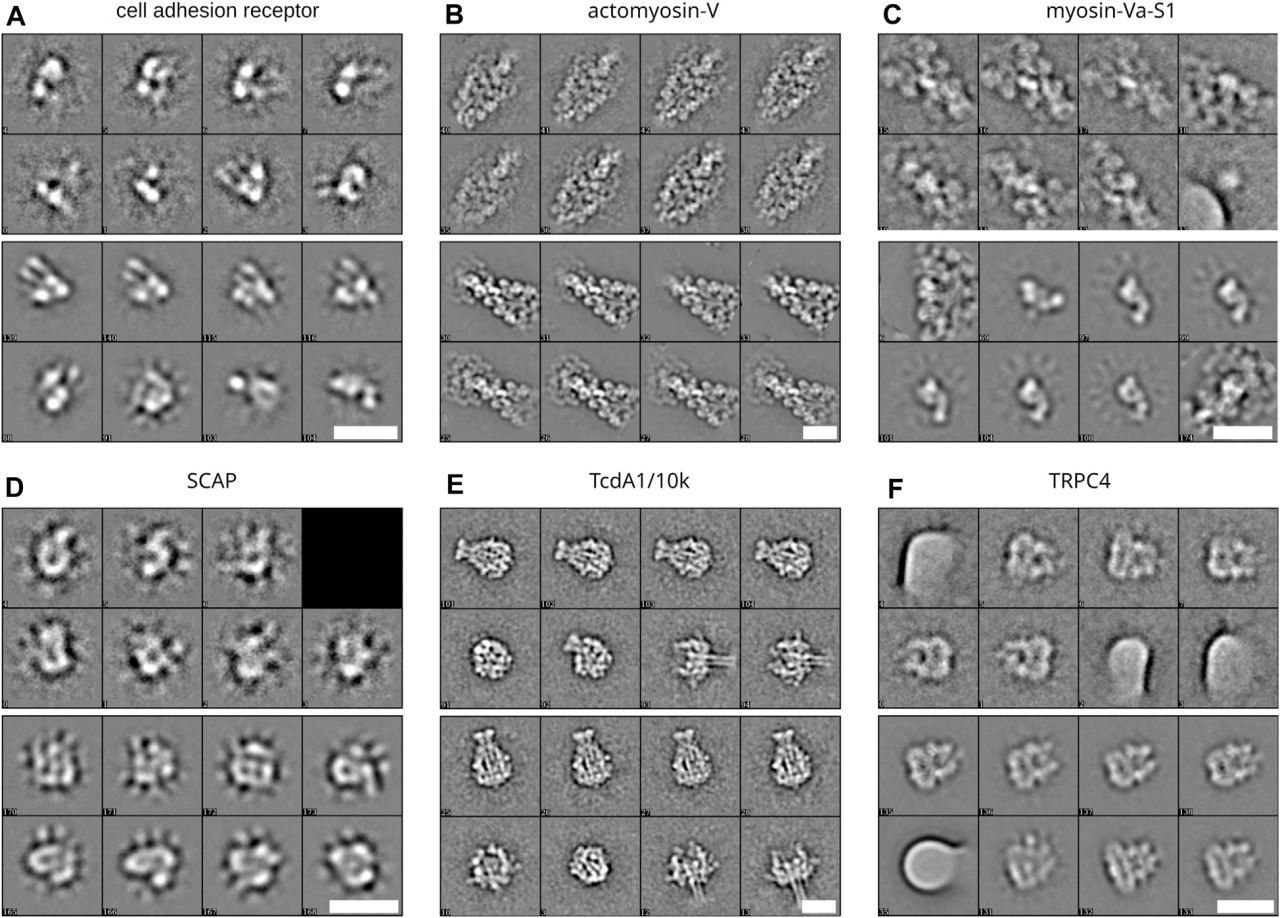
Class averages from different data sets. Averages produced when processing the different data sets in Figure 3 **(A–F)** Each section depicts a block of eight representative averages when processing 10,000 particles (top eight averages) and 200,000 particles (lower eight averages) of cell adhesion receptor, actomyosin-V, myosin, SCAP, TcdA1/10k, and TRPC4, respectively. Scale bars, 20 nm.

Above we have shown the total runtime of GPU ISAC to scale linearly with the size of the input stack. Next to the capabilities of the used GPU hardware, the overall quality of the data set will be the prime factor affecting the slope of the linear function approximating the runtime scaling. To demonstrate the differences in total runtime required for different data sets, we processed six data sets on the same hardware platform. For each data set, we processed subsets containing 10,000, 50,000, 100,000, and 200,000 particles, respectively. When plotting the total runtime we can show that processing more data results in linear scaling of the total runtime, with some variation depending on the data (Figure 3A). The most difficult to process data set (CAR) also exhibits the highest variance in its linear processing trend. Regardless of the data, the vast majority of the overall runtime can be seen to be spent in the iterative clustering steps of the ISAC algorithm (Figure 3B). As above, while processing more data generally results in more classes, the amount of this increase in returns diminishes the more particles are added (Figure 3C). The produced averages can be seen to feature a much higher SNR, depending on how many particles were processed (Figure 4).

#### 3.1.4 Processing Large-Scale Data Sets (1M+)

**FIGURE 5:**
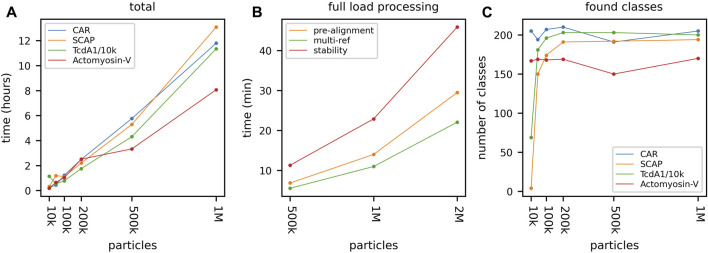
CUDA kernel heavy load processing. Processing four data sets containing up to two million particles using two RTX 6000 cards. **(A)** Total processing time can be seen to scale linearly with the input size and processing of one million particles was completed within 8–12 h. **(B)** To demonstrate the performance of the GPU ISAC CUDA kernels we show the time needed to process 500,000, 1,000,000, and 2,000,000 particles of actomyosin-V, respectively. Shown are processing times during the first ISAC iteration only, as this is the only time when the ISAC algorithm processes the full stack and no particles have been rejected, yet. Note that the pre-alignment includes five repetitions of full-stack alignment, and stability testing includes five repetitions of thirty times aligning all data with their designated class average, each. As is shown, the GPU ISAC CUDA kernels managed to repeatedly align 2,000,000 particles 150 times (stability testing) in less than 40 min. **(C)** By default, GPU ISAC is configured to provide 200 classes. As can be seen, once more than 100,000 particles are being processed, GPU ISAC produces a full set of averages.

To examine the performance of the newly developed CUDA kernels, as well as stress-test the new data batching mechanisms, we processed four large-scale data sets containing one and two million particles and extracted the relevant time stamps from the automatically generated GPU ISAC log messages. At its core, the ISAC algorithm operates in iterations, where, at the end of each iteration, any particles that could not be accounted for during the clustering step are discarded from future processing. Because of this, the GPU ISAC CUDA kernels will process the full input stack only during the very first ISAC main iteration. In addition, the three GPU-accelerated bottleneck functions—pre-alignment, multireference alignment, and stability testing—are being applied iteratively as well. More specifically, the pre-alignment is repeated 14 times; multireference alignment is applied during each of five k-means clustering repetitions per iteration; and stability testing involves five repetitions of intra-class alignments, each of which consists of aligning all particles with their class average 30 times. In other words, the presented timing results denote the time to stream and process the data on the available GPUs multiple times—more than a hundred times, in fact, in the case of stability testing. To produce large-scale timing results, we processed one million particles of actomyosin-V, SCAP, CAR, and a repeated set of TcdA1 (Figure 5A). We processed multiple in order to demonstrate the overall scaling, and recorded the timings required by the CUDA kernels to process the data during the first ISAC main iteration. To process these large data sets, we used two high-performance Quadro RTX 6000 cards. As shown, these cards allow us to process one million particles in about seven to fourteen hours. In addition, the most time-consuming GPU-accelerated function of stability testing repeatedly processed one million particles in about 25 min, and two million particles in less than 50 min (Figure 5B). These results also demonstrate the overall linear scaling property of GPU ISAC when using the same parameters across multiple runs processing different amounts of particles. Across data sets, the pre-set limit of equal-sized classes is reached when using 200,000 particles. The only exception in this case is actomyosin-V, where the number of averages stays constant regardless of the number of used particles (Figure 5C). The produced averages feature a high signal to noise ratio except in the case of processing TcdA1/10, which contains the same subset of 10,000 particles 100 times (Supplementary Figure S3).

### 3.2 Comparison With Cluster Node Parallelization

A direct comparison between the existing (cluster/CPU) implementation ISAC2 and the newly developed GPU ISAC is difficult, as running ISAC2 on a single machine is not what ISAC2 was developed for. However, we can approximate the number of processes in our locally available cluster that is required to match the performance of GPU ISAC on a single machine. Each cluster node is equipped with high end Intel Xeon Gold 6134 CPUs, each providing 16 MPI processes. To present our comparison, we processed 500,000 particles of actomyosin-V on twelve cluster nodes (192 MPI processes) and measured the overall runtime on four available GPU platforms: Two GV-100 cards, two GTX 1080 TI cards, four GTX 1080 TI cards, and two RTX 6000 cards. As can be seen, already a single machine using only two GTX 1080 TI cards processed the data slightly faster than twelve cluster nodes. Four GTX 1080 TI cards required 65% of the clusters processing time, and two RTX 6000 cards only required 60% of the time spent by the twelve cluster nodes (Supplementary Figure S4). Regardless of the used ISAC implementation, the produced averages feature the same quality (Supplementary Figure S5)**.**

### 3.3 GPU Iterative Stable Alignment and Clustering Scaling Behavior

While the size of the input data set is a fundamental parameter determining the overall runtime, it is usually not a parameter changed by the user—once a data set is obtained (and cleaned), there is little reason not to use all particles available. Other than the size of the input stack, the most relevant user-accessible parameters to determine ISAC runtime are the maximum and minimum amount of particles used to form individual classes. These numbers indirectly determine how many ISAC iterations are required in order to process a data set at hand. Sorting into more, smaller classes is more effort than sorting into fewer, larger classes. Unfortunately, the ISAC algorithm itself cannot deduce the optimal class size for a given data set. It can, however, provide linear runtime scaling across input dimensions in order to make sure that determining the correct parameter values and producing a larger number of classes does not become prohibitively expensive. We have already seen above that processing larger input stacks while using default parameters yields a linear scaling of overall runtime (Supplementary Figure S1). Note that this also implies linear scaling with increasing class size, as by default GPU ISAC will always attempt to produce 200 classes—processing more data consequently also means producing larger classes. In the following we go through the remaining ISAC input dimensions, examine the runtime behavior when parameters values are increased, and present the resulting scaling behavior across both data and hardware platforms.

#### 3.3.1 Scaling With Number of Particles and Number of Classes

At the very core of the ISAC algorithm operates an equal-sized k-means clustering function that ensures that no class contains more than a pre-set number of particles. Choosing the correct maximum (and minimum) class size imposes a limit on the number of classes that ISAC will attempt to produce and consequently significantly influences the runtime behavior. While the optimal value will always depend on the particular data set at hand, we can nevertheless examine the impact of modifying this parameter across multiple data sets. To do so, we processed increasingly larger substacks of the actomyosin-V data set, while fixing the number of particles allowed within any individual class. This results in GPU ISAC producing more equal-sized classes when given more particles to process (Supplementary Figure S6D). Of the three GPU-accelerated bottleneck functions, both the pre-alignment and the stability test alignment can be seen to scale almost perfectly linearly (Supplementary Figure S6A,C). The multireference alignment accompanying the k-means iterations, however, has to not only deal with a larger data volume, but also a higher number of classes with which to align every particle. This increase in two input dimensions simultaneously results in quadratic scaling behavior (Supplementary Figure S6B).

#### 3.3.2 Scaling With Number of Particles per Class

For practical purposes, the number of particles when processing a data set is probably the most relevant parameter value to adjust when using GPU ISAC to produce clean class averages. As stated above, by default GPU ISAC will attempt to produce 200 classes and adjust the maximum class size accordingly when reading the size of the input stack. In order to determine the impact of choosing a different class size we repeatedly processed a myosin-Va-S1 data set containing 200,000 particles using a maximum class size of 250, 400, 500, and 1,000, respectively. When profiling the runtime spent in the GPU-accelerated bottleneck function we can see that this choice is irrelevant for the pre-alignment, since this function is called before any classes have been determined (Supplementary Figure S7A). The multireference alignment step requires more processing time the smaller the class size and, consequently, the higher the number of classes (Supplementary Figure S7B). The stability test alignments remain mostly constant, as this function depends much more on the overall number of particles being processed, as described above (Supplementary Figure S7C). Although an increase in class size generally results in an increase in runtime, this correlation exhibits much more variance than our other metrics (Supplementary Figure S7D). This variation depends on the processed data set and is a result of the number of main iterations GPU ISAC requires to successfully cluster the data into stable classes—the higher the signal to noise ratio throughout the data, the more reliable this correlation. Finally, as expected, a smaller class size yields a higher number of classes given no increase in the number of processed particles (Supplementary Figure S7E).

## 4 Discussion

In this study, we present GPU ISAC: A computationally more efficient version of the successful 2D clustering ISAC algorithm that foregoes its predecessor’s requirement for a computer cluster, and instead provides high quality class averages on a single machine. By employing any locally available GPUs as co-processors, GPU ISAC uses a newly developed set of CUDA kernels to execute the bottleneck computations of the original ISAC algorithm. A new set of batch mechanisms divides the overall data into individual work-load packages that are distributed evenly to the available GPUs. This dynamic work-load allocation during runtime enables GPU ISAC to process data sets of essentially arbitrary size.

Our timing and benchmarking results show that GPU ISAC is able to process data sets containing millions of particles overnight, and reliably sort them into classes that produce clean class averages. Further, the class resolution of ISAC allows for underrepresented sub-populations present in the data to be assigned their own classes and be clearly visible in the overall set of class averages. By providing accurate class assignments, GPU ISAC can also be used to clean a given data set on a single machine by identifying the particles that contribute to stable, clean classes, and, by extension, those particles that do not. This allows users to reject unusable particles and thereby enable any computationally expensive follow-up processing to only focus on the subset of particles that actually contributes to computing a high resolution structure.

Comparing the total runtime of GPU ISAC processing data on a single machine with ISAC2 processing the same data on a cluster, we showed that using only two GTX 1080 TI consumer-grade cards is already enough to outperform twelve high end cluster nodes (192 processes). Using high performance RTX 6000 cards, GPU ISAC processing time is further lowered to only 60% of this cluster benchmark line. Note that increasing the number of cluster nodes also increases the amount of RAM available to ISAC2 and, consequently, a large enough cluster will always outpace a single GPU-equipped computer. However, the single GPU machine can be stored in an office without additional cooling, can be acquired for a fraction of the funds, can be set up within a week, and requires a minimum amount of maintenance when compared to a full-scale cluster.

In addition to enabling ISAC clustering on single machines, the overall user experience is further enhanced by improved user feedback and status messages. Additional sanity checks and relative default parameters eliminate the most commonly encountered issues when performing ISAC clustering. Improved error handling enables users to identify possible problems more easily with their data set and/or the chosen parameters. A new standalone installer includes a miniature example that can be used to confirm successful operation before a more elaborate run is performed, and further integrates GPU ISAC into an existing SPHIRE installation, allowing the program to be configured and executed from within the SPHIRE GUI.

The results above show that large data sets can be processed efficiently by GPU ISAC using consumer-grade graphics hardware. While the exact processing time will always depend on the convergence behavior of the data set at hand, our results show that the total runtime scales linearly with the input size. This holds especially for our newly developed CUDA kernels performing the bottleneck alignment computations: Processing times scale almost perfectly linear across almost all tested parameter variations. This includes using larger data sets, producing more classes, and producing larger classes—in each case the user does not have to worry about a sudden increase in processing time when adapting these parameters to the needs of the data set at hand. This linear scaling further ensures GPU ISAC to be future-proof in the face of ever-growing data sets: Rather than operating in the acceptable range of quadratic or even exponential scaling, linear scaling allows GPU ISAC to comfortably keep pace with more data simply by linearly upscaling its computational resources—such as replacing current GPUs with their consumer-grade equivalent of the following hardware generation.

The fact that GPU ISAC enables us to compute high quality averages on a single machine also allows us to integrate efficient 2D clustering into existing workflows and/or be combined with other local processes. As an example, GPU ISAC was integrated in the TranSPHIRE (Stabrin et al., 2020) pipeline, where it was combined with deep learning tools to enable on-the-fly cryo-EM reconstructions during data acquisition.

## Data Availability

The raw data supporting the conclusions of this article will be made available by the authors, without undue reservation.
