# Supplementary material for: Accelerated 2D Classification With ISAC Using GPUs
Source: Front Mol Biosci. 2022 Jul 6;9:919994. doi: 10.3389/fmolb.2022.919994 (PMC9296836; doi:10.3389/fmolb.2022.919994)
Supplement: Supplementary file 1 [file DataSheet1.ZIP › supplementary_information/schoenfeld_et_al_gpu_isac_supplementary_figures.docx]

**
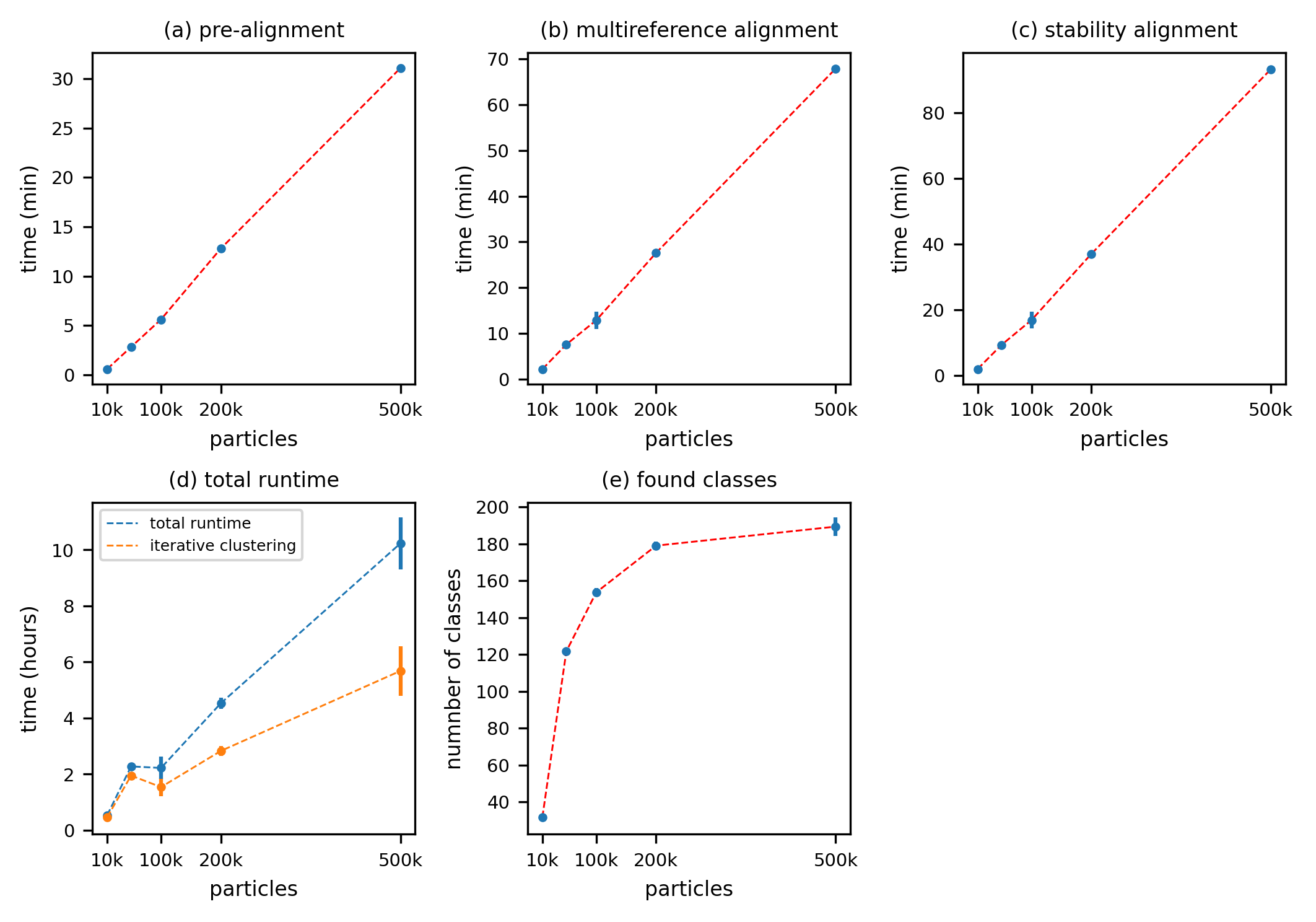
**

**Supplementary Figure 1. GPU ISAC general performance.** We repeatedly processed subsets of the myosin-V-S1 data set using two GV-100 GPUs. Subsets contained 10,000, 50,000, 100,000, 200,000, and 500,000 particles, respectively. Shown are the accumulated amounts of time spent by the three GPU-accelerated bottleneck computations when executing a full GPU ISAC run: **(a)** The one-time pre-alignment, **(b)** the multireference alignment used after each clustering iteration, and **(c)** the numerous, repeated alignments performed during stability testing once clustering has concluded. In each case, plots show the average values of three repeated runs using the same input data and parameters. **(d)** Since cryo-EM data are memory intensive, the initial file handling and pre-processing of the data can be quite costly. To show this, we plotted the total runtime **(stippled blue)** as well as the actual processing time spend in ISAC proper **(stippled orange)**, after the initial file-reading operations and other once-performed pre-processing steps have concluded. The GPU computation time of all bottleneck computations scale almost perfectly linearly with the input size, where the slope of the linear function depends on the capabilities of the used GPU hardware. In addition, this observed linear scaling still holds when processing data sets that cannot fit into GPU memory and have to be processed in batches. In other words, the additionally introduced data transfer overhead has no perceptible effect on the linear scaling performance of the GPU ISAC CUDA kernels. **(e)** Numbers of found classes. Note that due to using the default parameters, GPU ISAC will not produce more than 200 classes here.

**
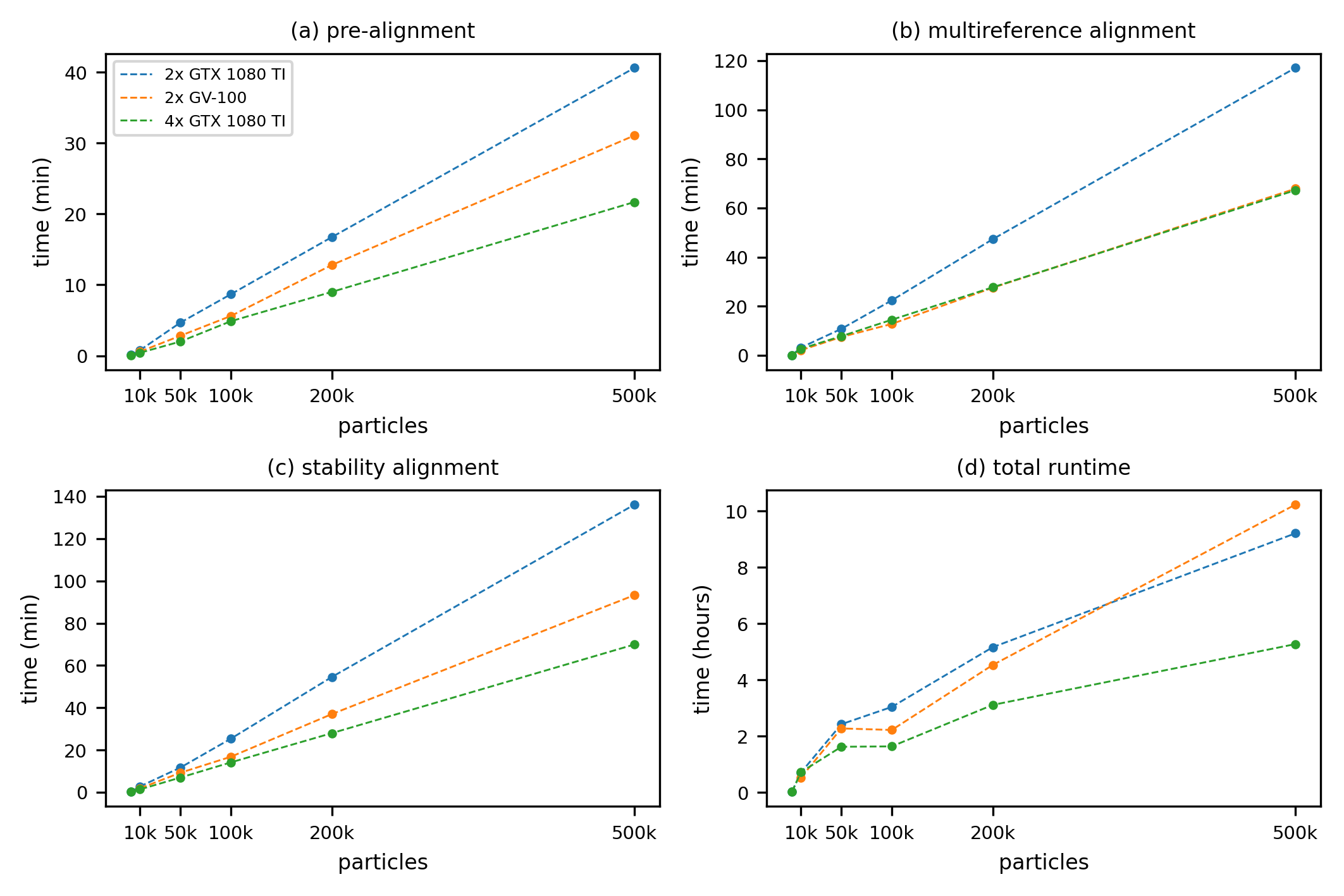
**

**Supplementary Figure 2. GPU ISAC using different GPU platforms.** We processed subsets of the myosin-V-S1 data set containing 500,000 particles using three different GPU arrangements: Two GeForce GTX 1080 TI cards **(blue)**, two GV-100 cards **(orange)**, and four GeForce GTX 1080 TI cards **(green)**. On each GPU setup we processed subsets of the overall data set, consisting of 10,000, 50,000, 100,000, and 500,000 particles, respectively **(x-axis)**. **(a)** Shown are the accumulated processing times of the three GPU-accelerated bottleneck computations of running the one-time pre-alignment, **(b)** the once-per-clustering multireference alignment, and **(c)** the repeated alignments of the ISAC stability test. **(d)** Total runtime of the different runs is shown.

**
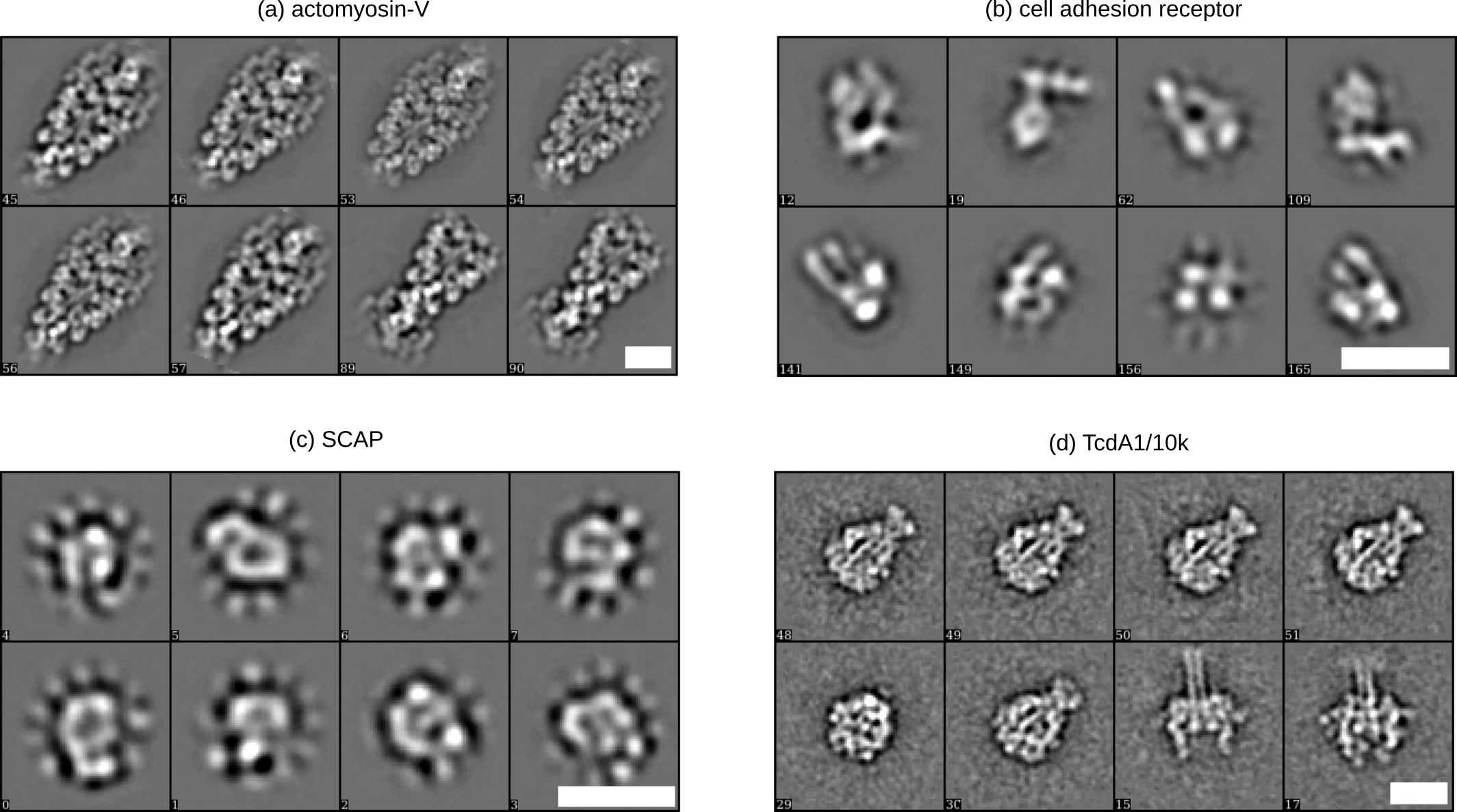
Supplementary Figure 3. Averages from 1,000,000 particles. (a-d)** Eight representative averages produced when processing 1,000,000 particles of actomyosin-V, cell adhesion receptor, SCAP, and TcdA1/10k, respectively. Scale bars, 20 nm.

**
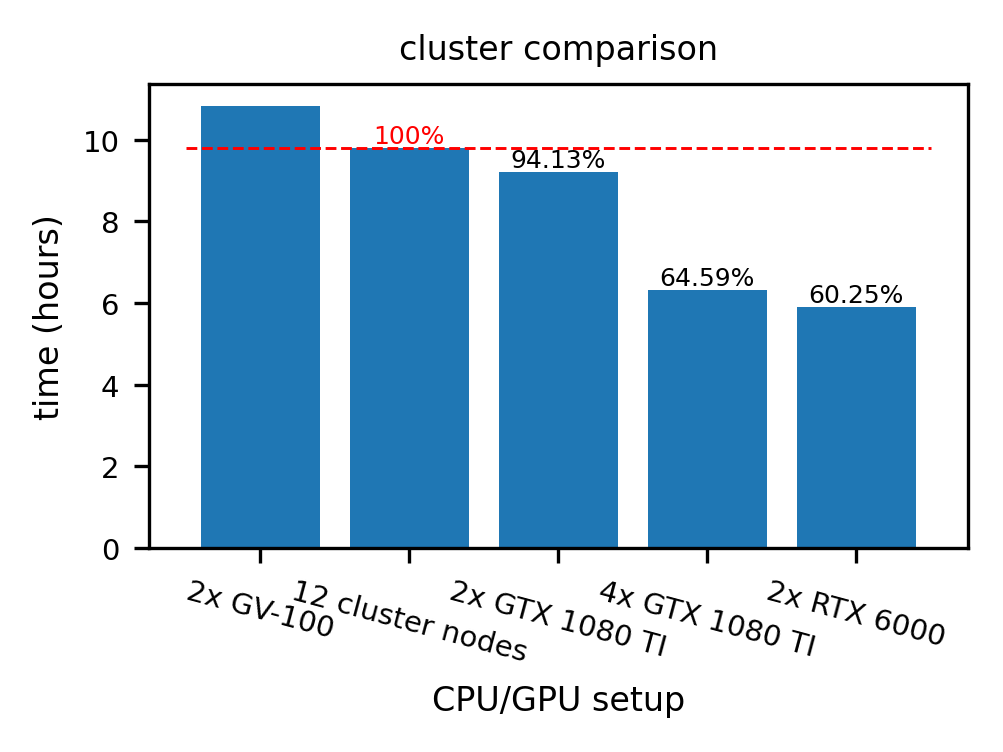
**

**Supplementary Figure 4. Comparison with cluster nodes.** To demonstrate the number of cluster nodes that we can replace by using GPU ISAC, we processed 500,000 actomyosin-V particles using ISAC2 on 12 nodes of our local cluster, and four different GPU platforms. Each cluster node houses a high-performance Intel Xeon Gold 6134 CPU and provides 16 MPI processes, for a total of 192 processes. Cluster node performance is denoted as 100% time consumption (stippled red) and faster GPU ISAC runtimes are annotated with a relative percentage of the cluster reference performance. Not included in the cluster performance is the waiting time before the run was actually executed after the job was submitted to the queue.

**
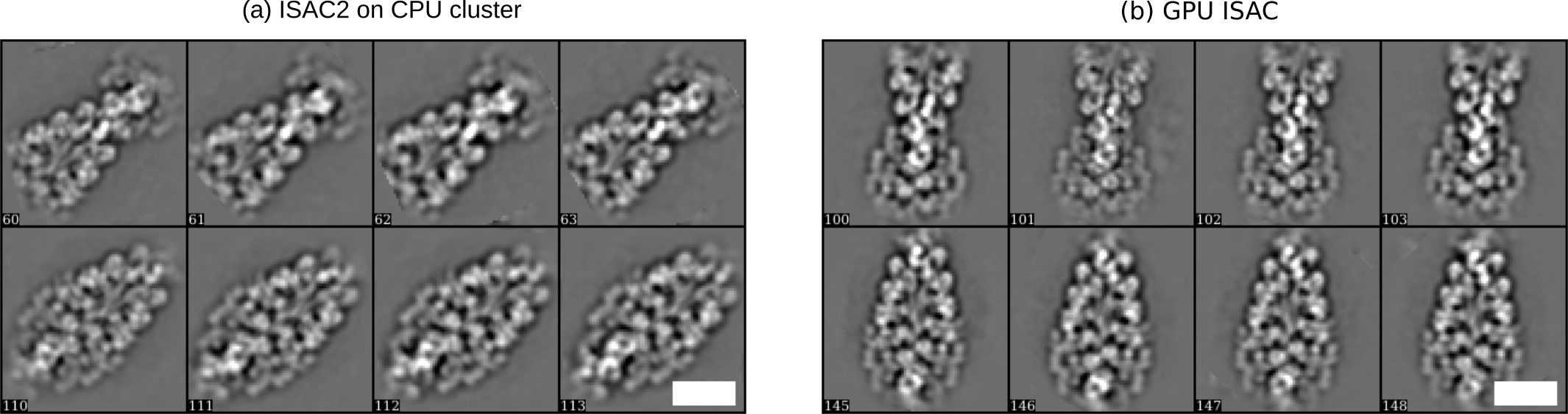
**

**Supplementary Figure 5. Average comparison between ISAC implementations. (a)** Set of eight representative averages when processing 500,000 actomyosin-V particles using ISAC2 on a cluster using 12 nodes. **(b)** Set of eight representative averages when processing the same data using GPU ISAC on a single machine. Scale bars, 20 nm

**
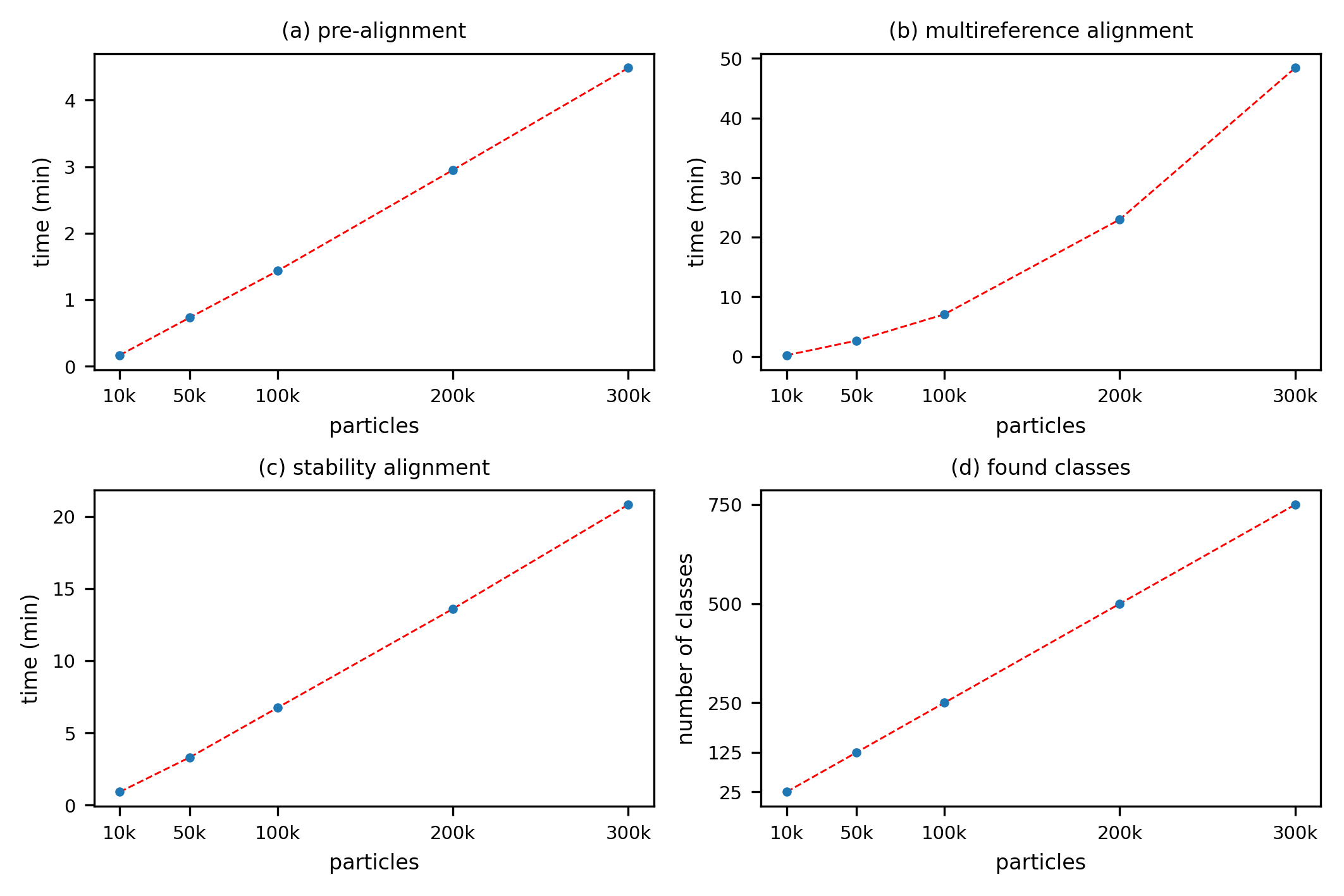
**

**Supplementary Figure 6. Increasing the number of classes.** To determine the scaling behavior with an increased amount of classes, we processed increasingly larger subsets of a data set containing 300,000 actomyosin-V particles. Across all runs the maximum class size was kept fixed at the same value, resulting in GPU ISAC processing and sorting the available particles into an increasing number of equal-sized classes. Plotted is the accumulate time consumption of the three GPU-accelerated bottleneck functions. **(a)** At the time of the initial pre-alignment no class designations exist yet, and consequently this functions scales perfectly linear as shown before. **(b)** Multireference alignment can be seen to no longer scale linearly when processing a larger number of equal-sized classes. This behavior is caused by the multireference function aligning every available particle with every available class. Consequently, a simultaneous increase in both the number of particles as well as the number classes results in a quadratic scaling behavior. **(c)** For each class, the stability testing repeatedly aligns the particles of the class with its own class average. The data volume to be processed by this function grows linearly, and so does the time consumption of the responsible GPU-accelerated CUDA kernel. **(d)** Each run produces the full set of class averages, confirming that each run indeed processed the expected amount of classes.

**
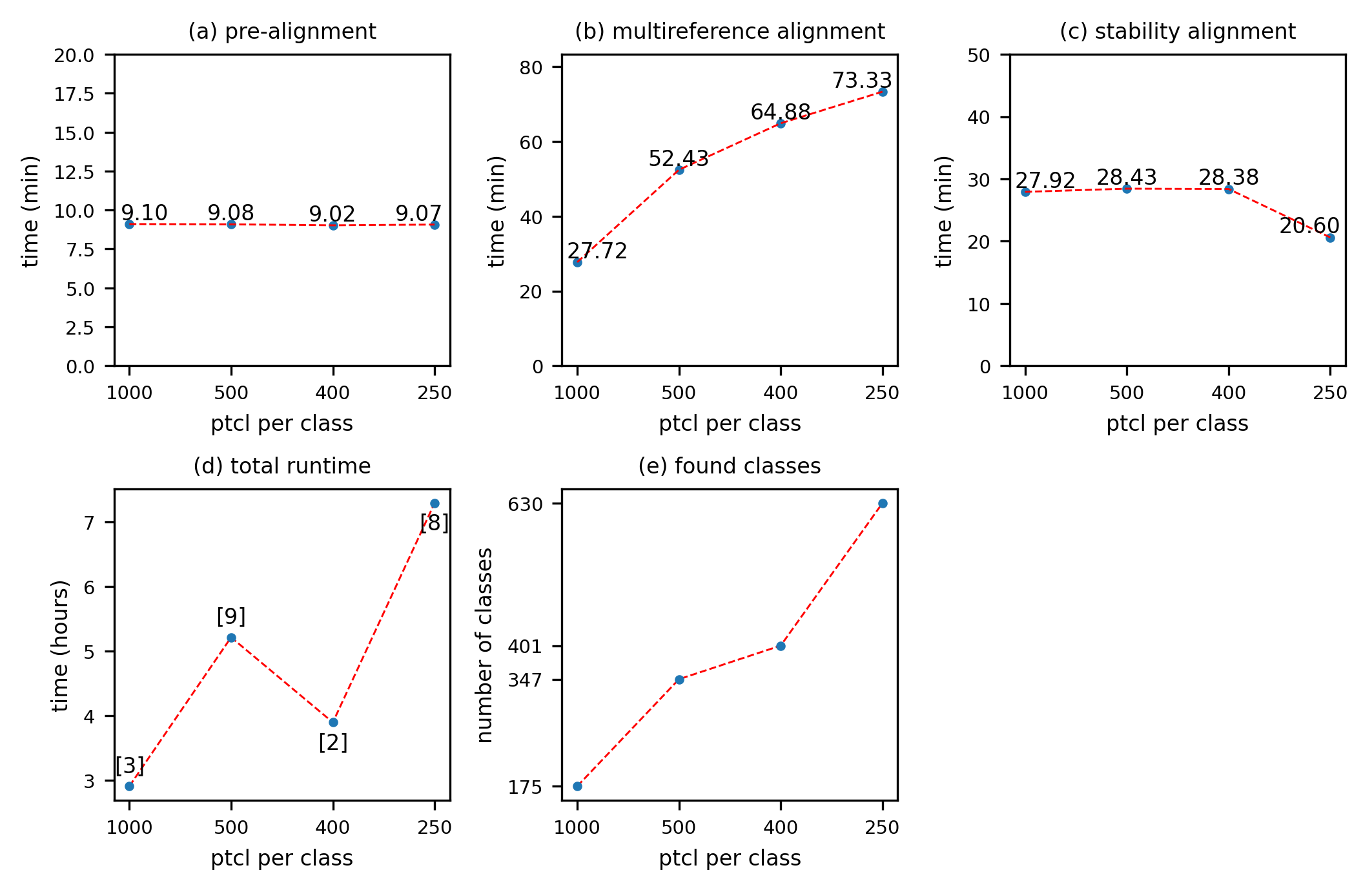
**

**Supplementary Figure 7. Number of particles per class.** To determine the impact of this key parameter, we repeatedly processed a myosin-Va-S1 data set containing 200,000 particles. The runs were configured to fill classes with 1,000, 500, 400, and 250 particles, respectively. Bar plots are annotated with their precise runtime (y-values). **(a)** Since the amount of processed particles stayed constant across these runs, the pre-alignment tales almost exactly the same time in each case. **(b)** During clustering, a smaller class size results in the overall input stack getting partitioned into a larger number of classes. Since the multireference alignment aligns all particles with all available classes, this takes more time as the number of classes increases. **(c)** During the stability alignment the workload stays on a similar level. **(d)** In general, runtime is higher when more classes need to be determined, but this correlation shows a much higher degree of variance than our other metrics, as does the number of main iterations required for processing (annotated in parentheses). **(e)** The number of found classes, predictably, rises with a smaller maximum class size. Note, however, that dividing the number of particles by the class size merely gives an upper bound of the number of produced classes.
